# YTHDF1 promotes breast cancer progression by facilitating FOXM1 translation in an m6A-dependent manner

**DOI:** 10.1186/s13578-022-00759-w

**Published:** 2022-02-23

**Authors:** Hengyu Chen, Yuanhang Yu, Ming Yang, Haohao Huang, Shenghui Ma, Jin Hu, Zihan Xi, Hui Guo, Guojie Yao, Liu Yang, Xiaoqing Huang, Feng Zhang, Guanghong Tan, Huangfu Wu, Wuping Zheng, Lei Li

**Affiliations:** 1 Department of Breast and Thyroid Surgery, The Second Affiliated Hospital of Hainan Medical University, Key Laboratory of Tropical Translational Medicine of Ministry of Education & Hainan Provincial Key Laboratory of Tropical Medicine, Haikou, 570311 China; 2 Department of Breast and Thyroid Surgery, Union Hospital, Tongji Medical College, Huazhong University of Science and Technology, Wuhan, 430022 China; 3 Department of Pancreatic Surgery, Union Hospital, Tongji Medical College, Huazhong University of Science and Technology, Wuhan, 430022 China; 4 Department of Neurosurgery, General Hospital of Central Theater Command of Chinese People’s Liberation Army, Wuhan, 430070 China; 5 Medical College Wuhan University of Science and Technology, Wuhan, 430070 China; 6 Department of Emergency Medicine, Affiliated Hospital of Sergeant School Affiliated to Army Medical University, Shijiazhuang, 050047 China

**Keywords:** Breast cancer, YTHDF1, FOXM1, m6A, EMT

## Abstract

**Background:**

N6-methyladenosine (m^6^A) is the most common post-transcriptional modification at the RNA level. However, the exact molecular mechanisms of m6A epigenetic regulation in breast cancer remain largely unknown and need to be fully elucidated.

**Methods:**

The integrating bioinformatics analyses were used to screen clinical relevance and dysregulated m6A “reader” protein YTHDF1 in breast cancer from TCGA databases, which was further validated in a cohort of clinical specimens. Furthermore, functional experiments such as the CCK-8 assay, EdU assay, wound healing assay, transwell invasion assay and cell cycle assay were used to determine the biological role of YTHDF1 in breast cancer. RIP, m6A-IP, and CLIP assays were used to find the target of YTHDF1 and further verification by RT-qPCR, western blot, polysome profiling assay. The protein–protein interaction between YTHDF1 and FOXM1 was detected via co-immunoprecipitation.

**Results:**

Our study showed that YTHDF1 was overexpressed in breast cancer cells and clinical tissues specimens. At the same time, the high expression level of YTHDF1 was positively correlated with tumor size, lymph node invasion, and distant metastasis in breast cancer patients. YTHDF1 depletion repressed the proliferation, invasion and epithelial-mesenchymal transformation (EMT) and induced G0/G1 phase cell cycle arrest of breast cancer cells in vitro and in vivo. We also demonstrated that FOXM1 is a target of YTHDF1. Through recognizing and binding to the m6A-modified mRNA of FOXM1, YTHDF1 accelerated the translation process of FOXM1 and promoted breast cancer metastasis. Whereas overexpression of FOXM1 in breast cancer cells partially counteracted the tumor suppressed effects caused by YTHDF1 silence, which further verified the regulatory relationship between YTHDF1 and FOXM1.

**Conclusion:**

Our study reveals a novel YTHDF1/FOXM1 regulatory pathway that contributes to metastasis and progression of breast cancer, suggesting that YTHDF1 might be applied as a potential biomarker and therapeutic target. That also advances our understanding of the tumorigenesis for breast cancer from m6A epigenetic regulation.

**Supplementary Information:**

The online version contains supplementary material available at 10.1186/s13578-022-00759-w.

## Background

m^6^A modification is the most abundant RNA modification in eukaryotic cells [1], and mainly targets messenger RNA (mRNA) and non-coding RNA (ncRNA). m^6^A modification is reversible, and the methylation and demethylation processes were performed by specific methyltransferases (writers) and demethylases (erasers), respectively [2]. m^6^A modification does not change the sequence of RNA base. It regulates gene expression by affecting the structure of RNA to alter its interaction with m^6^A binding protein (readers) or other protein complexes. m^6^A modification occurs in more than 30% of mRNAs in mammals, with an average of 3–5 m^6^A modifications per mRNA [3]. m^6^A modification is essential for regulating gene expression at multiple levels (transcription, post-transcription, translation and post-translation) and influencing critical physiological and pathological processes [3]. The physiological processes affected by m^6^A modification mainly include DNA repair, differentiation of hematopoietic stem cells, the development of tissues and embryos, etc. In addition, the abnormal expression of the writers, erasers and readers is also an important cause of tumorigenesis [4–8].

YTH N6-Methyladenosine RNA Binding Protein 1 (YTHDF1) is a member of the YT521-B homologous (YTH) domain protein family [9, 10]. It is highly conserved in evolution, and it regulates gene expression by binding to m^6^A-modified mRNAs through its C-terminal YTH domain, thereby changing the fate of genes [10, 11]. Although the YTH family members have similar functional domains, they have different regulatory effects on the target mRNA. The role of YTHDF2, the first reader to be identified, is to guide the mRNAs to the decay sites (such as the processing body) to induce their degradation [12, 13]. YTHDF1, on the other hand, promotes the translation of m^6^A-modified mRNAs by interacting with transcription initiation factors [14, 15]. When the YTH proteins are abnormally expressed, the degradation and translation of oncogenes or tumor suppressor genes are disorganized, and tumorigenesis is triggered [16].

Recent studies have shown that YTHDF1 is highly expressed and associated with poor prognosis in various tumor tissues such as colon cancer [17], melanoma [18], liver cancer [19], and non-small cell lung cancer [20]. In breast cancer, although it has been reported that YTHDF1 expression is significantly increased in tumor tissues compared with para-cancerous tissues and is closely associated with poor prognosis [21], its specific mechanisms have not yet been clarified.

In the present study, we found that YTHDF1 was aberrantly expressed in breast cancer tissues by analyzing the cancer genome atlas (TCGA) database and further verified this finding via breast patient samples and cell lines. Moreover, patients with high YTHDF1 expression had poor overall survival (OS), and this survival difference was more distinct in the HER2+ subgroup. Data from our clinical specimens indicated that YTHDF1 was also positively correlated with tumor size, lymph node invasion as well as distant metastasis in breast cancer patients. Subsequently, a series of functional experiments confirmed that YTHDF1 promoted the proliferation and invasion of breast cancer cells in vitro and in vivo. In brief, we demonstrated that YTHDF1 binds to the m^6^A-modified FOXM1 gene at the coding sequence (CDS) region and plays its role as an oncogenic gene by promoting FOXM1 translation rather than transcription, exacerbating the progression of breast cancer.

## Materials and methods

### Patient samples and cell lines

Breast cancer and para-cancerous tissues of 48 patients were collected from the Department of Breast and Thyroid Surgery, Union Hospital, Tongji Medical College, Huazhong University of Science and Technology from June 2019 to December 2020. After careful examination of the tissues by three pathologists, the patients were diagnosed with breast cancer. Patients who received any treatment (including surgery, chemotherapy, immunotherapy, etc.) before the surgery were excluded from the study. Informed consent was signed by all patients. The study was approved by the Ethics Committee of Tongji Medical College, Huazhong University of Science and Technology. The study was in accordance with the Declaration of Helsinki.

The cell lines used in the study included human normal breast cell line MCF-10A, human breast cancer cell line MCF-7, MDA-MB-231, SKBR3, T47D and BT-549. MCF-10A, MCF-7, MD-MB-231, T47D and BT-549 were purchased from American Type Culture Collection (ATCC). SKBR3 was purchased from China Center for Type Culture Collection (CCTCC). Cells except for MCF-10A were cultured in DMEM medium supplemented with 10% FBS (Gibco, US) and antibiotics (Penicillin 100 U/ml, Streptomycin 100 mg/ml) (Gibco, US). For MCF-10A, the culture conditions were: mixture of DMEM and F12 medium (1:1) (Gibco, US), antibiotics (Pen/Strep), 5% horse serum (Ausbian, AUS), insulin (10 µg/ml) (Thermo Fisher Scientific, US), epidermal growth factor (20 ng/ml) (Gibco, US), choleramycin (100 ng/ml) (Sigma, US) and hydrocortisone (0.5 µg/ml) (Tocris, US). Cells were cultured at 37 ℃ with 5% CO_2_. Cells were cultured for up to 20 passages.

### Cell proliferation assay

Cell proliferation was measured by CCK-8 (Cell Counting Kit-8, Dojindo, Japan) and EdU assays. For the CCK-8 assay, 1 * 10^3^ cells were seeded and cultured in 96-well plates for 24 h, 48 h, 72 h, 96 h and 120 h; 10 µl CCK-8 solution was added to each well. After adding the solution, the culture was continued for 4 h, and the absorbance was measured every 30 min using a microplate reader at 450 nm.

EdU proliferation assay was carried out using Click-iT Plus EdU Alexa Fluor 594 flow cytometry assay kit (Thermo Fisher Scientific, Catalog No. C10646) according to the manufacturer's instructions. Images were captured by the confocal microscope (Olympus, Japan) at ten randomized fields.

### Cell cycle assay

2 * 10^5^ cells were seeded in a 12-well plate for 48 h, and then cell cycle assays were performed. The procedures are briefly described: cells were collected by centrifugation at 8500 rpm for 5 min, and 70% cold ethanol was added to the cells dropwise. Cells were then fixed at 4 °C for 30 min. Next, wash the cells with 1× Phosphate Buffer Saline (PBS) twice. After staining the cells with propidium iodide (PI) antibody for 30 min, the cell cycle was measured by flow cytometry.

### Cell invasion assay

Breast cancer cell invasive ability was measured by transwell insert chambers (Corning, NY, USA) pre-coated with Matrigel (Corning Inc.). In brief, 2 * 10^4^ cells were seeded into the upper chamber containing 200 µl serum-free medium, and 500 μl culture medium with 20% FBS was added into the bottom chamber. After 24 h, invading cells were fixed with 4% paraformal-dehyde and stained with 0.1% crystal violet. Cells were counted using a microscope for 5 fields randomly at 100× magnification.

### Immunohistochemistry and HE staining

Hematoxylin and eosin (HE) staining was conducted following the standard protocol. For immunohistochemistry staining, tissue slides (6 μm) were deparaffinized and hydrated. Antigen retrieval was performed using citrate solution (PH 6), and the tissues were then blocked with 2% BSA for 2 h at room temperature. Next, tissues were incubated overnight with primary antibody (anti-YTHDF1: Abcam, catalog No. 230330) at 4 °C. Subsequently, tissues were incubated with HRP-conjugated antibody for at least 1 h in the darkroom at room temperature. Images were captured with the microscope (Leitz, Italy), and the results were analyzed using ImageJ software (version 1.8.0, NIH, US). The IRS (immunoreactive score) scoring system to determine immunohistochemical positivity and the protein expression levels. IRS = SI (Staining Index) * PP (Proportion of Positive tumor cells). SI ranges from 0 to 3 and PP from 0 to 4. IRS score ranges from 0 to 12. For protein expression, IRS 0–1: no expression, 2–3: low expression, 4–8: moderate expression, 9–12: high expression [22]. The mean integrated optical density (IOD) values of YTHDF1 and FOXM1 in tumor tissues were calculated by Image Pro-Plus 6.0 software (MEDIA CYBERNETICS, US).

### RNA immunoprecipitation (RIP)

RIP was carried out to detect mRNAs that bind to the YTHDF1 protein. The RIP was conducted using a Megna RNA-binding protein kit (Millipore, US). 10 µl YTHDF1 or IgG antibody (Cell signaling Technology, US) was incubated with protein A/G magnetic beads for at least 4 h at 4 °C. After lysing the cells with RIP buffer, the cells were incubated with the washed magnetic beads at 4 °C overnight. Then RNA was extracted by Trizol reagent (Invitrogen, US), and the expression of the target genes was detected by RT-qPCR.

### Cross-linking immunoprecipitation (CLIP)

The CLIP assay was conducted according to the protocol as previously described [23]. Briefly, 2 * 10^7^ MCF-7 cells were irradiated with the UV crosslinker (Thermo Fisher Scientific, US) (wavelength 260 nm). The cells were then lysed with the RIPA buffer and co-incubated with protein A/G magnetic beads at 4 °C for at least 4 h. The immune complexes were then eluted at 60 °C for 20 min with a 50 mM Tris–HCl solution (pH 7.8). To isolate the RNA from the immune complexes, we use chloroform to extract RNA. Finally, RT-qPCR was used to detect and analyze the expression level of the target genes.

### m^6^A immunoprecipitation (m^6^A-IP)

1.5 µg of IgG or m^6^A antibody was conjugated to protein A/G beads at 4 °C overnight. Next, incubate 300 µg fragmented RNA with the IgG or m^6^A antibody in the immunoprecipitation buffer containing RNase inhibitor at 4 °C overnight. RNA was eluted from the beads using elution buffer and was extracted for RT-qPCR by phenol and ethanol.

### Polysome profiling

Polysome profiling was conducted according to the protocols described previously [24]. After treated with 0.1 mg/ml cycloheximide for 10 min, cells were washed with cold PBS and lysed with polysome lysis buffer (10 mM Tris–HCl, pH 7.4; 10 mM MgCl_2_; 0.3 mM NaCl; 1% Triton X-100; RNase inhibitor and 100 µg/ml cycloheximide). Cell lysates were centrifuged at 2000 rpm for 5 min and 12,000 rpm for 15 min. RNA concentration was detected by Nanodrop (Thermo Fisher Scientific, US). 500 µg RNA was then added in freshly prepared sucrose gradient solutions (10–50%) and centrifuged at 30,000 rpm for 4 h at 4 °C. 1 ml fractions were collected. Total RNA was extracted by RNeasy Mini Kit (QIAGEN, US) for qRT-PCR analysis.

### Lentivirus production and cell transduction

HEK-293 T cells were used to produce lentivirus particles. Briefly, pLKO.1 puro-sh-YTHDF1#1/sh-YTHDF1#2 or pLKO.1 puro control vector, helper vector pxPAX2 and envelope vector pMD2.G were transfected into HEK-293 T cells using Lipofectamine 2000 (Thermo Fisher Scientific, US). Supernatants containing lentivirus particles were collected and filtered after 48 h transfection. 1 * 10^4^ cells were incubated with 1 ml supernatants for 24 h and were selected using 4 μg/ml puromycin. 1 μg/μl puromycin was used as a selective pressure to get stably transfected cell lines. sh-YTHDF1#1 and sh-YTHDF1#2 were synthesized by GenePharma (CN). Oligo sequence of sh-YTHDF1#1: 5′-CGGTGGGACAAATGTGAACAT-3′; sh-YTHDF1#2: 5′-CCCGAAAGAGTTTGAGTGGAA-3′; sh-YTHDF1-3: 5′-GTTCGTTACATCAGAAGGATA-3′. sh-FOXM1#1: 5′-GCTGGGATCAAGATTATTA-3′; sh-FOXM1#2: 5′-GGCTGCACTATCAACAATA-3′. Scramble shRNA was purchased from Addgene (#1864, US).

### Cell transfection

Transfection of plasmids was performed using Lipofectamine 2000 reagent (Invitrogen) according to the manufacturer's instruction. YTHDF1-WT (Addgene #70087), YTHDF1-MUT, FOXM1-WT (Addgene #68811, FOXM1 isoform 3) and FOXM1-MUT, CCNB1 (Addgene #39871) overexpression plasmids were used for transfection. Detailed information on the mutations was shown in Additional file 2: Fig. S2. The sequences of siRNAs targeting YTHDF1 (si-YTHDF1) were as follows: siYTHDF1-1: 5′-GAACAAAAGGACAAGAUAAUA-3′, si-YTHDF1-2: 5′-CAAAAGGACAAGAUAAUAAAG-3′.

### RT-qPCR

Total RNA was extracted from cells using TRIzol Reagent (Life Technologies) according to the manufacturer's instructions. RNA was transcribed into cDNA using the Reverse Transcription Kit (Takara, JP). RT-qPCR was conducted by SYBR Green Master Mix (#4309155, Applied Biosystems, US). The procedure was set as follows: Hold 95 °C for 10 min; Denature 95 °C 15 s and Anneal/Extend 60 °C 1 min for 40 cycles. Primers were listed in Additional file 5: Table S1.

### Western blot and antibodies

Cells were washed with cold Phosphate Buffered Saline (PBS, Thermo Fisher Scientific, US) twice before protein extraction. Total proteins were extracted from cells using RIPA buffer (Sigma Aldrich, US) supplemented with the protease inhibitor cocktail (Roche, Shanghai, CN) according to the manufacturer's instructions. 25–30 μg proteins were loaded onto the 4–12% SDS-PAGE (BeyoGel, CN). Electrophoresis was performed at 120 V for 1 h. Proteins were then transferred to the PVDF membranes at 250 mAh for 2 h. After blocking with 5% non-fat milk for 30 min, membranes were incubated with primary antibodies overnight at 4 °C. Western blot images were captured by a myECL imager (Thermo Fisher Scientific, US). Primary antibodies used in the present study were listed as follows: FOXM1 (A2493, ABclonal), YTHDF1 (17479-1-AP, Proteintech), ZEB1 (21544-1-AP, Proteintech), N-cadherin (22018-1-AP, Proteintech), E-cadherin (20874-1-AP, Proteintech), Vimentin (10366-1-AP, Proteintech), Snail (ab216347, Abcam), β-Actin (ab8226, Abcam), anti-Flag tag (SAB4301135, Sigma-Aldrich), anti-HA-tag (05-904, Sigma-Aldrich).

### In vivo assays

Animal Experiments were conducted under the approval of the ethics committee of Tongji Medical College, Huazhong University of Science and Technology (Protocol #TJU 2018-0025). NOD/SCID immune-deficient mice were purchased from Shanghai Experimental Animal Center. 2 * 10^6^ MCF-7 cells transduced with sh-NC or sh-YTHDF1-were subcutaneously injected into the mice (5/group). Tumor width and length were measured every 7 days. Tumor volume = (length * width^2^)/2. After 7 weeks, mice were sacrificed, and the weight of tumors was detected. Xenografts were collected for HE staining, immunohistochemistry staining and western blot analysis.

For spontaneous lung metastasis assay, 4 * 10^6^ sh-NC or sh-YTHDF1#2 transduced MCF-7 cells were injected into the mammary fat pads of the NOD/SCID mice (5/group). The primary tumor was removed when its volume reached 150 mm^3^. The mice were sacrificed, and lung metastasis nodules were counted 12 weeks after the removal.

### Statistical analysis

Data were expressed as mean ± standard derivation (SD). A T-test was used for comparison between two groups. A one-way ANOVA test followed by an SNK test was used for comparison between three groups. Kaplan–Meier curve was used for survival analysis. All data were analyzed using GraphPad Prism 9.0 (GraphPad Software, US). Statistical significance is considered as *P < 0.05, **P < 0.01, ***P < 0.001. All experiments were three independent repetitions.

## Results

### YTHDF1 is highly expressed in breast cancer samples and cells

**Fig. 1:**
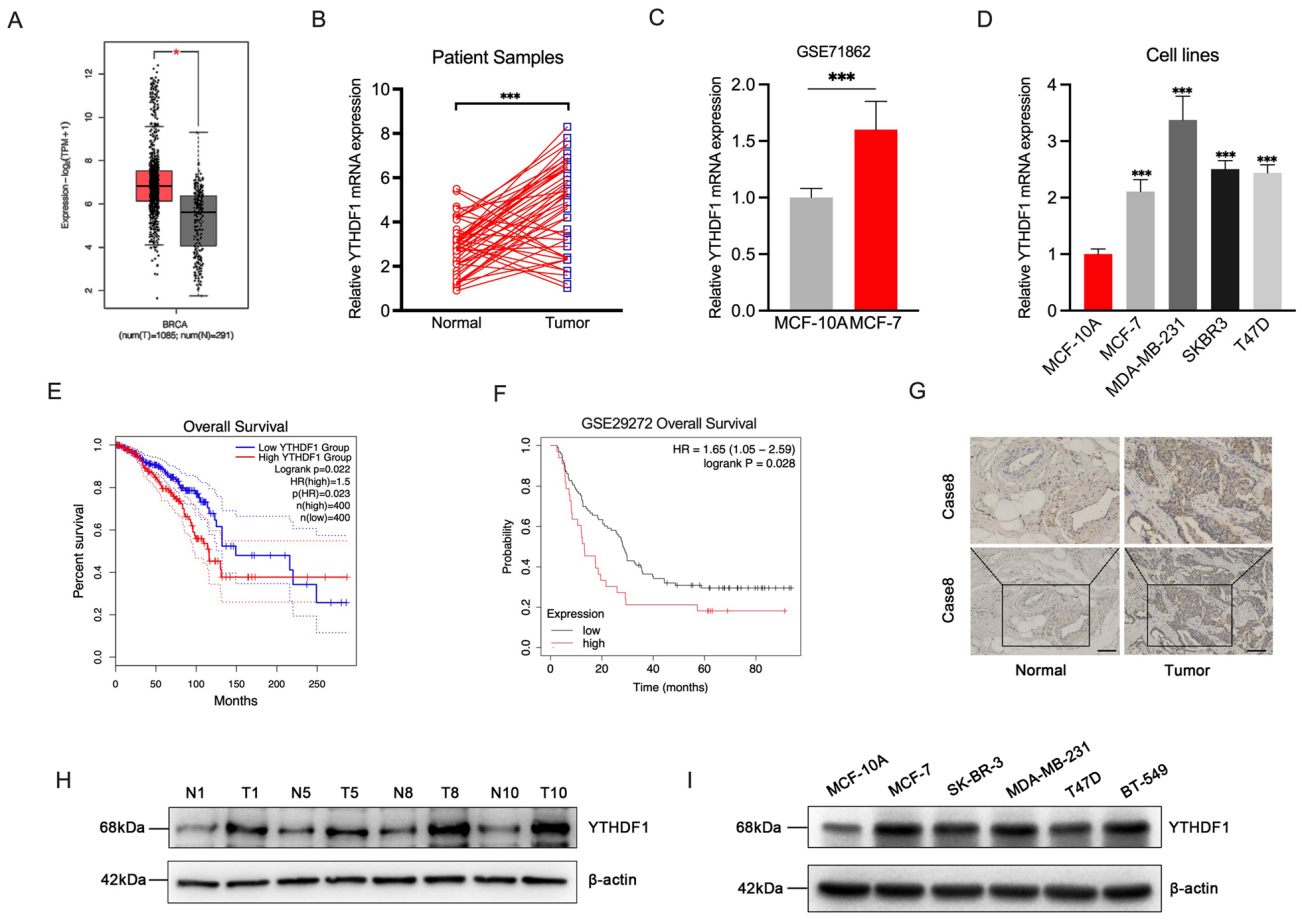
YTHDF1 was highly expressed in breast cancer samples and cells. **A** YTHDF1 expression in breast cancer tissues and normal tissues from TCGA-BRCA dataset. Relative YTHDF1 mRNA expression in **B** collected breast cancer tissues and adjacent normal tissues samples, **C** MCF-10A and MCF-7 cells from GSE71862 dataset and** D** normal human breast cell line MCF-10A and breast cancer cell line MCF-7, MDA-MB-231, SKBR3, T47D and BT-549. Kaplan–Meier curves of OS in the TCGA dataset **E** and GSE29272 dataset **F** of all breast cancer patients with high and low YTHDF1 expression. **G** Immunohistochemical staining of YTHDF1 in a breast cancer sample. YTHDF1 protein expression in **H** breast cancer tissues and adjacent normal tissues and **I** normal human breast cell line MCF-10A and breast cancer cell line MCF-7, MDA-MB-231, SKBR3, T47D and BT-549. All data were presented as means ± SD of at least three independent repetitions. Values are significant at *P < 0.05, **P < 0.01, ***P < 0.001 as indicated

To identify the protein related to m6A modification that potentially drives the tumorigenesis of breast cancer, we first explored the expression of m6A “reader' protein in the TCGA-BRCA dataset. The result showed that the expression of YTHDF1 in breast cancer samples was significantly higher than that in the normal control group, but there was no significant difference in the expression of YTHDF1 in breast cancer patients of different stages and subtypes (Fig. 1A, Additional file 1: Fig. S1A, B). Similar results were obtained in the collected breast cancer tissues and adjacent normal tissues validated by RT-qPCR, western blot analysis and immunohistochemical staining (Fig. 1B, G, H). We further explored the expression of YTHDF1 in cell lines. The results of the GSE71862 dataset showed that the expression of YTHDF1 in breast cancer cell line MCF-7 was 1.6 times that of human normal breast cell line MCF-10A (Fig. 1C). RT-qPCR and western blot analysis in the cell lines also suggested that the mRNA and protein expressions of YTHDF1 were significantly increased in the breast cancer cell lines (Fig. 1D, I). We also observed the effects of YTHDF1 on the prognosis of breast cancer patients. Kaplan–Meier analysis of TCGA-BRCA and GSE29272 datasets indicated that increased YTHDF1 was associated with shortened OS, which was a marker of poor prognosis in breast cancer (Fig. 1E, F). In addition, by stratification of TCGA-BRCA data, we also found that increased YTHDF1 could further distinguish the prognosis of the patients in the HER2+ and Luminal subtypes. Its high expression was significantly associated with poor prognosis (Additional file 1: Fig. S1C–E). To verify the clinical significance of YTHDF1 for breast cancer, we collected and examined its expression as well as the clinicopathologic features in a cohort of patients. The result indicated that the high expression level of YTHDF1 was positively correlated with tumor size, lymph node invasion, and distant metastasis in breast cancer patients (Additional file 5: Table S2). Taken together, these results demonstrate that YTHDF1 was dysregulated in breast cancer and that high expression of YTHDF1 was associated with poor outcomes in patients with breast cancer.

### YTHDF1 knockdown inhibits the growth ability and arrests the cell cycle of breast cancer cells

**Fig. 2:**
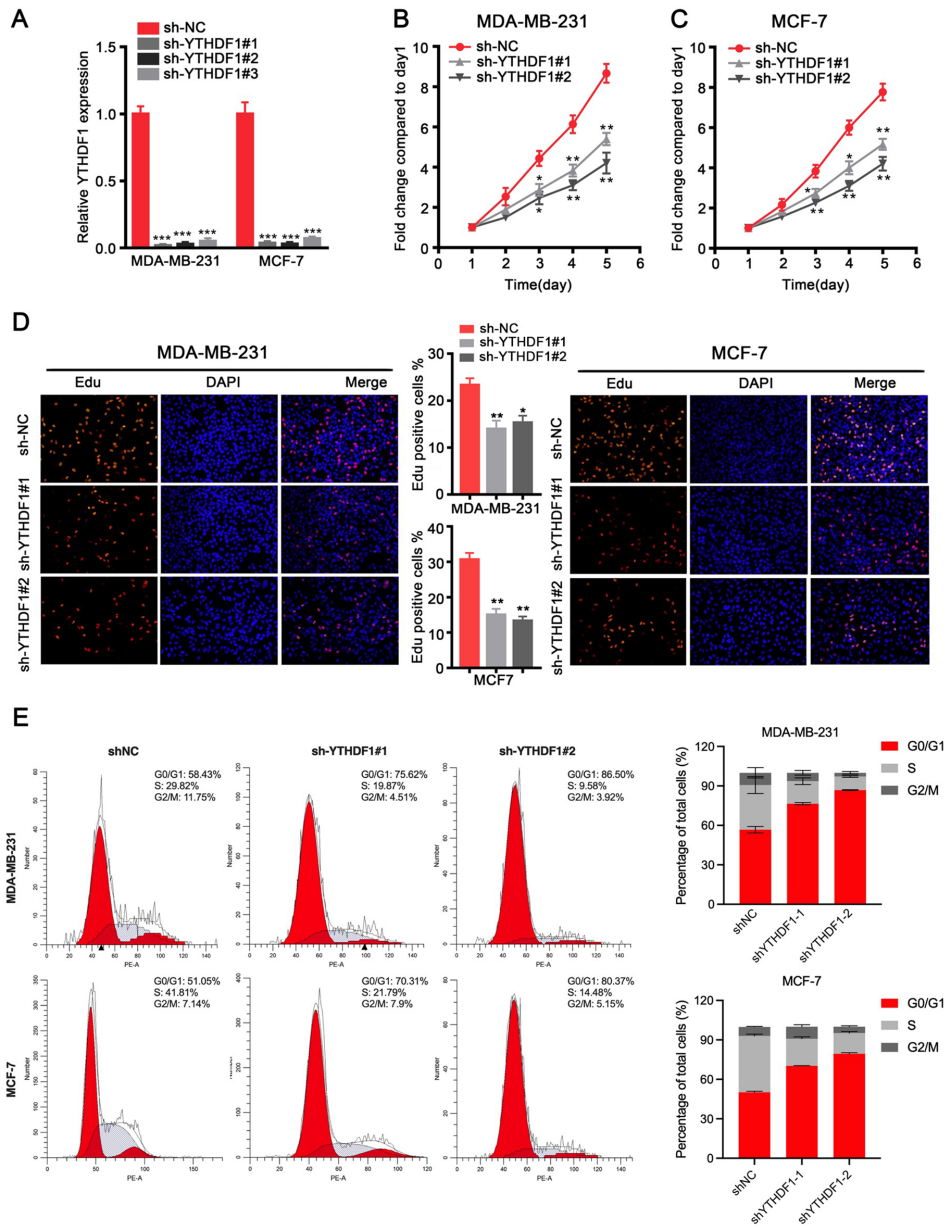
YTHDF1 knockdown inhibits the growth ability and arrests the cell cycle of breast cancer cells. **A** Relative YTHDF1 mRNA expression levels after transducing scramble shRNA (sh-NC), sh-YTHDF1#1, sh-YTHDF1#2 or sh-YTHDF1#3 in MDA-MB-231 or MCF-7 cells. **B** CCK-8 assay, **C** EdU proliferation assay and **D** cell cycle analysis in MDB-MB-231 or MCF-7 cells transduced with sh-NC, sh-YTHDF1#1 or sh-YTHDF1#2. All data were presented as means ± SD of at least three independent repetitions. Values are significant at *P < 0.05, **P < 0.01, ***P < 0.001 as indicated

We transduced shRNAs targeting YTHDF1 into breast cancer cell lines MDA-MB-231 and MCF-7 to knock down the expression of YTHDF1. After 48 h transduction, we examined the mRNA expression of YTHDF1 to verify the knockdown efficiency, and the results showed that all three shRNAs significantly suppressed the expression of YTHDF1 in MDA-MB-231 and MCF-7 cells (Fig. 2A). We then performed CCK-8 and EdU cell proliferation assays using scramble shRNA transduced MDA-MB-231 and MCF-7 cells (sh-NC) and sh-YTHDF1 transduced cancer cells. CCK-8 results indicated that the proliferation of cells in the sh-YTHDF1 group was significantly inhibited after 48 h incubation compared to the cells in the sh-NC group (Fig. 2B, C). EdU cell proliferation assay also showed that after 2 h incubation with the EdU medium, the proliferation of breast cancer cells in the sh-YTHDF1 group was significantly lower than that in the sh-NC group (Fig. 2D). In addition, we also investigated the effects of YTHDF1 on the cell cycle, and the results suggested that G0/G1 cell cycle arrest occurred in the sh-YTHDF1 group. (Fig. 2E).

### Silence of YTHDF1 restrains cell migration, invasion ability and EMT

**Fig. 3:**
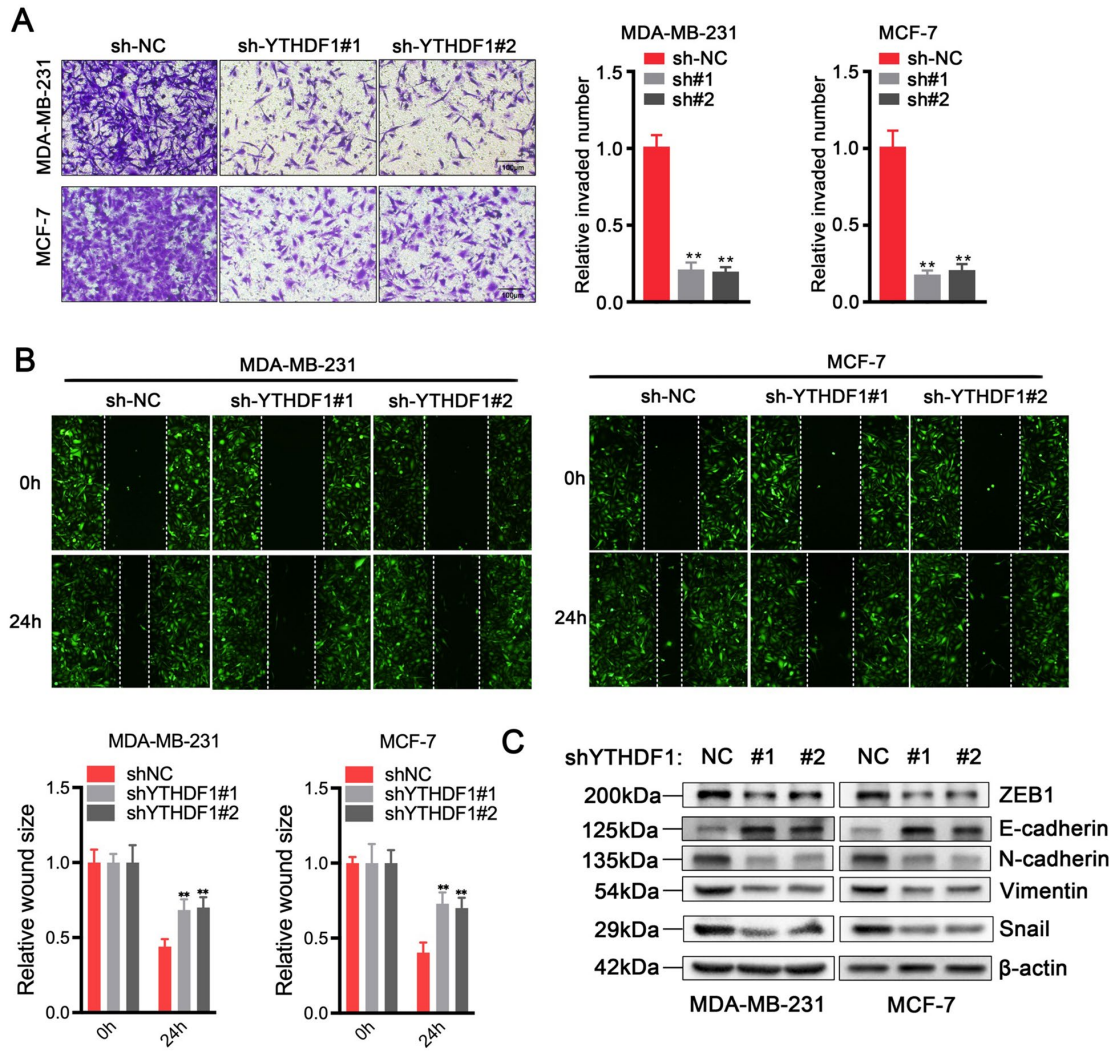
Silence of YTHDF1 restrain cell migration, invasion ability and EMT. **A** Transwell invasion assay, **B** wound healing assay and **C** western blot analysis for EMT-related proteins in MDA-MB-231 or MCF-7 cells transduced with sh-NC, sh-YTHDF1#1 or sh-YTHDF1#2 (sh#1:sh-YTHDF1#1; sh#2:sh-YTHDF1#2). All data were presented as means ± SD of at least three independent repetitions. Values are significant at *P < 0.05, **P < 0.01, ***P < 0.001 as indicated

We then investigated the role of YTHDF1 on the invasion capability of breast cancer cells. Transwell assay and wound healing assay revealed that knockdown of YTHDF1 could effectively suppress the migration and invasion ability of MDA-MB-231 and MCF-7 cells (Fig. 3A, B). Epithelial-mesenchymal transformation (EMT) was reported to be the first step of metastasis and played an essential role in breast cancer progression. Several studies have reported that YTHDF1 is involved in the molecular regulation pathways of EMT. Therefore, EMT-related proteins were examined by immunoblot assay, and the results showed that compared with the control group, the mesenchymal protein expressions of N-cadherin, Vimentin, ZEB1 and Snail in the sh-YTHDF1 group were significantly decreased. In contrast, epithelial marker protein E-cadherin was upregulated (Fig. 3C).

To ensure the accuracy of the experiment, similar experiments were carried out using si-YTHDF1 transfected MCF-7 cells. The results showed that after YTHDF1 knockdown with siYTHDF1, the proliferative and invasive ability of MCF-7 cells were significantly reduced, and the EMT was also inhibited (Additional file 3: Fig. S3).

### Depletion of YTHDF1 inhibits the growth of breast cancer cells in vivo

**Fig. 4:**
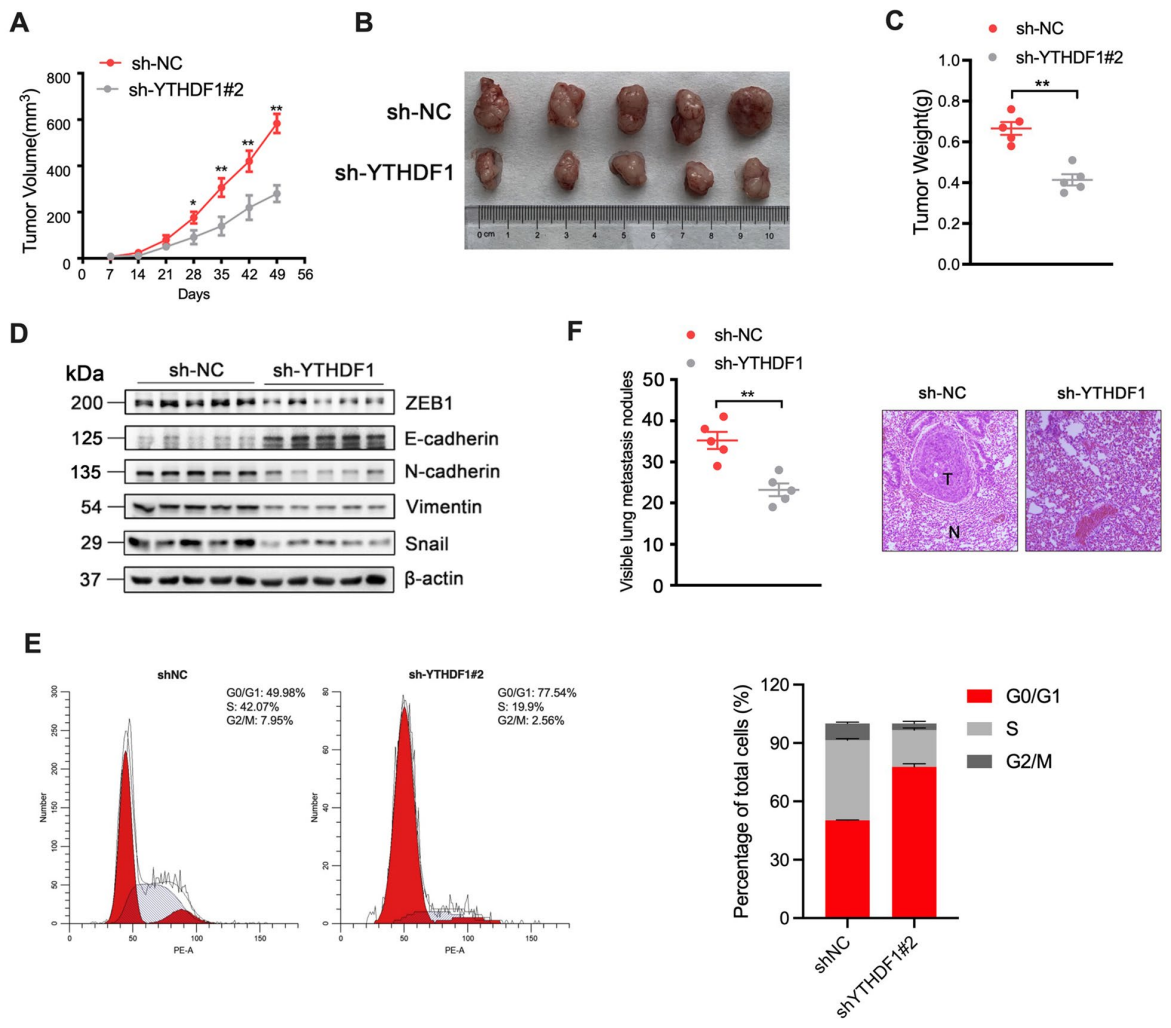
Depletion of YTHDF1 inhibits the growth of breast cancer cells in vivo. NOD/SCID mice (5 per group) were transplanted with MCF-7 cells (2 * 10^6^) transduced with sh-NC or sh-YTHDF1#2. **A** Tumor volumes in sh-NC or sh-YTHDF1#2 group at indicated days. **B** Representative images and **C** the weight of tumors in sh-NC or sh-YTHDF1#2 group at day 49. **D** The expression of EMT-related proteins and **E** cell cycle analysis in xenograft that transduced with sh-NC or sh-YTHDF1. **F** The number of visible lung metastasis nodules and representative images of lung HE staining slices. (T stands for metastasis and N for normal lung tissues). All data were presented as means ± SD of at least three independent repetitions. Values are significant at *P < 0.05, **P < 0.01, ***P < 0.001 as indicated

To further investigate the effect of YTHDF1 on breast cancer in vivo, MCF-7 cells stably transduced with a lentiviral vector containing negative control sequence or sh-YTHDF1#2 sequence were subcutaneously implanted into NOD/SCID mice. By calculating the tumor volume every 7 days, we found that the tumor volume in the sh-YTHDF1#2 group was significantly lower than that in the sh-NC control group since day28 (Fig. 4A). On day49, the mice were killed to measure the tumor weight, and it was found that the tumor weight of mice in the sh-YTHDF1#2 group was significantly lower than that in the sh-NC group (Fig. 4B, C). Western blot analysis and cell cycle assay of the tumor cells from xenograft showed that YTHDF1 silence could inhibit EMT in vivo and induce G0/G1 phase cell cycle arrest of breast cancer cells (Fig. 4D, E). Furthermore, in the lung metastasis assay, the number of metastatic lung nodules in the sh-YTHDF1#2 group was significantly less than that in the sh-NC group (Fig. 4F). Collectively, the above results imply that YTHDF1 could promote breast cell proliferative and invasive capacity both in vitro and in vivo. At the same time, the knockdown of YTHDF1 can significantly inhibit EMT in breast cancer.

### FOXM1 is a direct target of YTHDF1

**Fig. 5:**
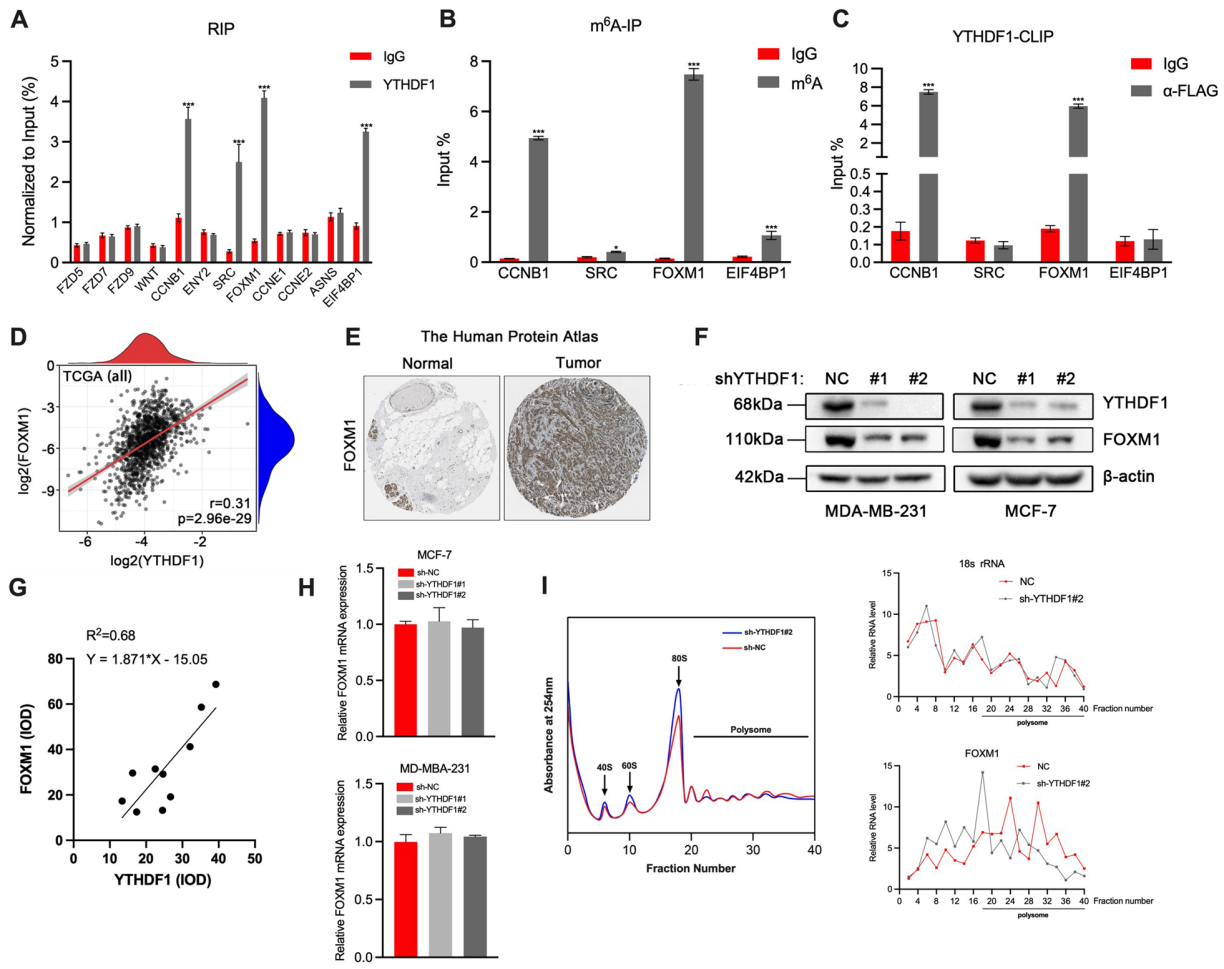
FOXM1 is a direct target of YTHDF1. Normalized expression of target genes detected by RT-qPCR after **A** RIP, **B** m6A-IP or **C** YTHDF1-CLIP assay. **D** Correlation between FOXM1 protein expression and YHTDF1 mRNA expression in TCGA-BRCA dataset. **E** FOXM1 protein expression in breast cancer tissue and normal tissue from The Human Protein Atlas. **F** The protein and expression of YTHDF1 and FOXM1 in MDA-MB-231 and MCF-7 cells transduced with sh-NC, sh-YTHDF1#1 or sh-YTHDF1#2. **G** The correlation between YTHDF1 and FOXM1 in protein levels by immunohistochemical analysis in 9 samples. **H** The mRNA expression of YTHDF1 and FOXM1 in MDA-MB-231 and MCF-7 cells transduced with sh-NC, sh-YTHDF1#1 or sh-YTHDF1#2. **I** Polysome profiling assay in MCF-7 cells transduced with sh-NC or sh-YTHDF1#2 (Left). FOXM1 and 18 s rRNA expression in different polysome fractions (Right). All data were presented as means ± SD of at least three independent repetitions. Values are significant at *P < 0.05, **P < 0.01, ***P < 0.001 as indicated

YTHDF1 was reported to influence the translation process of the target genes with m^6^A modifications. Therefore, we used the cBioPortal tool to find the genes whose protein expression was correlated with YTHDF1 mRNA expression in the TCGA-BRCA dataset. Finally, we selected 12 genes as the targets for further study (8 genes with the highest Spearman's correlation coefficients (CCNB1, ENY2, SRC, FOXM1, CCNE1, CCNE2, ASNS and EIF4BP1) and 4 genes that were previously reported to be regulated by YTHDF1 (FZD5, FZD7, FZD9 and WNT5A). After RIP, m^6^A-IP and CLIP assays were performed on MCF-7 cells, RT-qPCR was used to detect the expression levels of target genes. We found that FOXM1 and CCNB1 were occupied by YTHDF1 and modified by m^6^A simultaneously (Fig. 5A–C). Since FOXM1 plays a vital role in EMT, we chose FOXM1. We validated the correlation between YTHDF1 and FOXM1 expression in breast cancer patients from TCGA by estimating the Pearson's correlation coefficient R. The result indicated a positive correlation (r = 0.31, *P* = 2.96e–29) between YTHDF1 and FOXM1 expression (Fig. 5D). In addition, we found that FOXM1 protein expression was highly expressed in breast cancer tissues in The Human Protein Atlas database (Fig. 5E). To study the correlation between YTHDF1 and FOXM1 in protein levels, immunohistochemical analysis was carried out in 9 samples. Through linear regression analysis, it was found that FOXM1 protein levels were positively correlated with YTHDF1, with a correlation coefficient R^2^ = 0.68 (Fig. 5G).

To explore whether YTHDF1 regulates FOXM1 expression and from which aspect to regulate its expression, RT-qPCR, western blot and polysome profiling analysis were performed on breast cancer cells transduced with sh-NC or sh-YTHDF1#2. The results showed that for FOXM1, there was no significant difference in the mRNA expression between the sh-NC and sh-YTHDF1#2 groups. However, a considerable decrease in the protein expression was observed in the sh-YTHDF1#2 group (Fig. 5F, H). As shown in Fig. 5I, inhibition of YTHDF1 slightly decreased the translation level of MCF-7 cells. However, YTHDF1 knockdown inhibited FOXM1 translation significantly more than 18S rRNA. We further conducted polysome profiling assays in MCF-7 cells transfected with YTHDF1-WT and YTHDF1-MUT plasmids. As shown in Additional file 4: Fig. S4, the translational level of YTHDF1 increased in YTHDF1-WT but not in YTHDF1-MUT MCF-7 cells, indicating that mutant YTHDF1 did not promote FOXM1 translation. These results suggest that as a target of YTHDF1, FOXM1 is not regulated by YTHDF1 via affecting its transcription but promoting FOXM1 translation in breast cancer cells.

### YTHDF1 regulates FOXM1 expression in an m6A-dependent manner

**Fig. 6:**
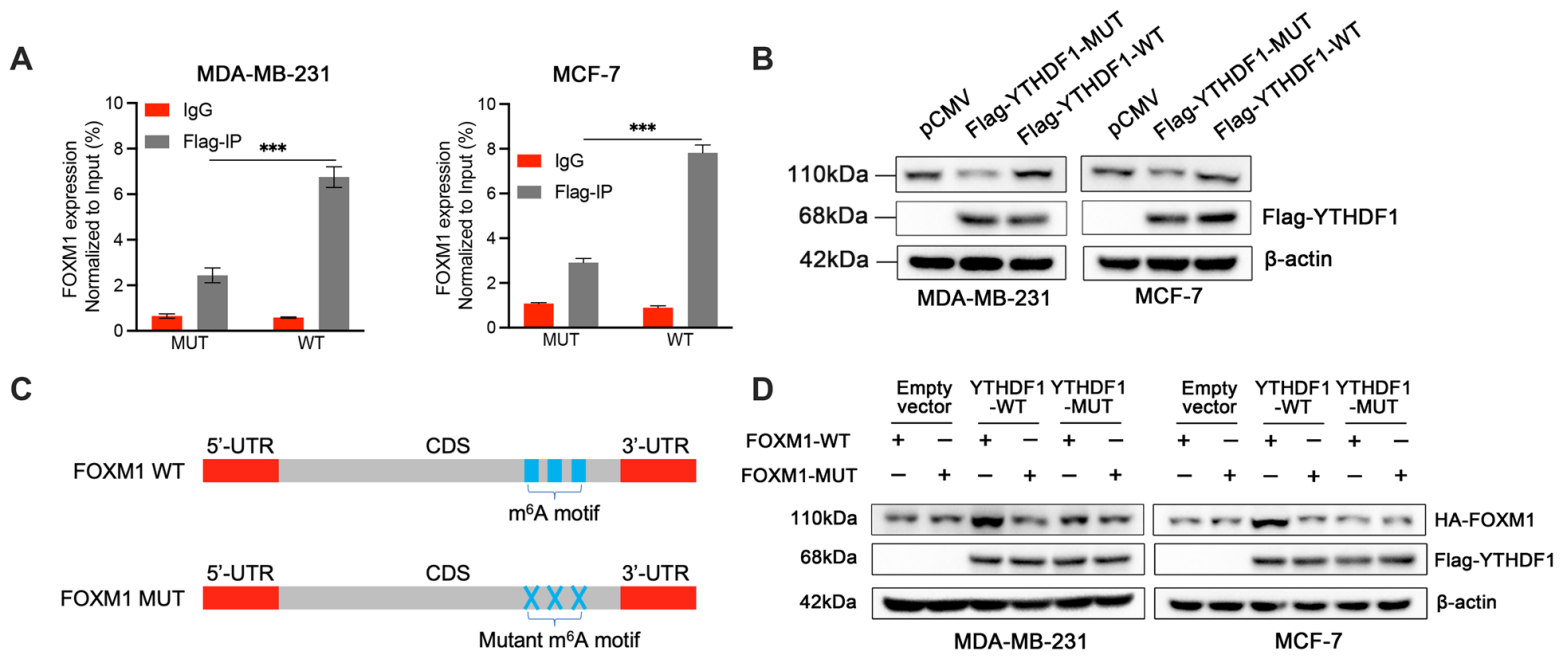
YTHDF1 regulates FOXM1 expression in an m^6^A-dependent manner. **A** The analysis of qPCR for FOXM1 expression in RIP assay (anti-Flag) in MDB-MA-231 and MCF-7 cells. **B** FOXM1 protein expression detected by western blot in MDB-MA-231 and MCF-7 cells transfected with YTHDF1-WT or YTHDF1-MUT plasmids. **C** Schematic image of FOXM1-WT plasmids, and FOXM1-MUT plasmids containing m^6^A motif mutations in the CDS region. **D** Protein expression of HA tagged FOXM1 in MDA-MB-231 and MCF-7 cells co-transfected with empty vector, YTHDF1-WT or YTHDF1-MUT, and FOXM1-WT or FOXM1-MUT plasmids. All data were presented as means ± SD of at least three independent repetitions. Values are significant at *P < 0.05, **P < 0.01, ***P < 0.001 as indicated

Since YTHDF1 can specifically identify the m6A modification and are responsible for the outcomes of its target genes, we then try to explore whether YTHDF1 promotes FOXM1 translation in an m^6^A dependent manner. Mutations of the YTH domain were introduced into the YTHDF1-WT plasmid to construct the YTHDF1-MUT plasmid (Additional file 2: Fig. S2A) [11, 25, 26]. As shown in Fig. 6A, B, an anti-Flag antibody was used for RIP assay, followed by RT-qPCR and western blot analysis to detect the expression of FOXM1. The results suggested that the binding of FOXM1 to YTHDF1 was significantly reduced in MD-MBA-231 and MCF-7 cells transfected with YTHDF1-MUT plasmids. Then we introduced mutations into the m6A motif of the FOXM1-WT plasmid to construct the FOXM1-MUT plasmid (Fig. 6C, Additional file 2: Fig. S2B). Subsequently, FOXM1-WT and FOXM1-MUT plasmids were co-transfected into the breast cancer cells with Empty vector, YTHDF1-WT and YTHDF1-MUT plasmids, respectively. The results suggested that the expression of FOXM1 was significantly increased only in the cells co-transfected with YTHDF1-WT and FOXM1-WT plasmids, while FOXM1-MUT had no response to the overexpression of YTHDF1-WT (Fig. 6D). In summary, these results confirmed that YTHDF1 modulated the translation process of FOXM1 in an m^6^A dependent manner.

### The inhibited effects of YTHDF1 silence could be rescued by FOXM1 overexpression

**Fig. 7:**
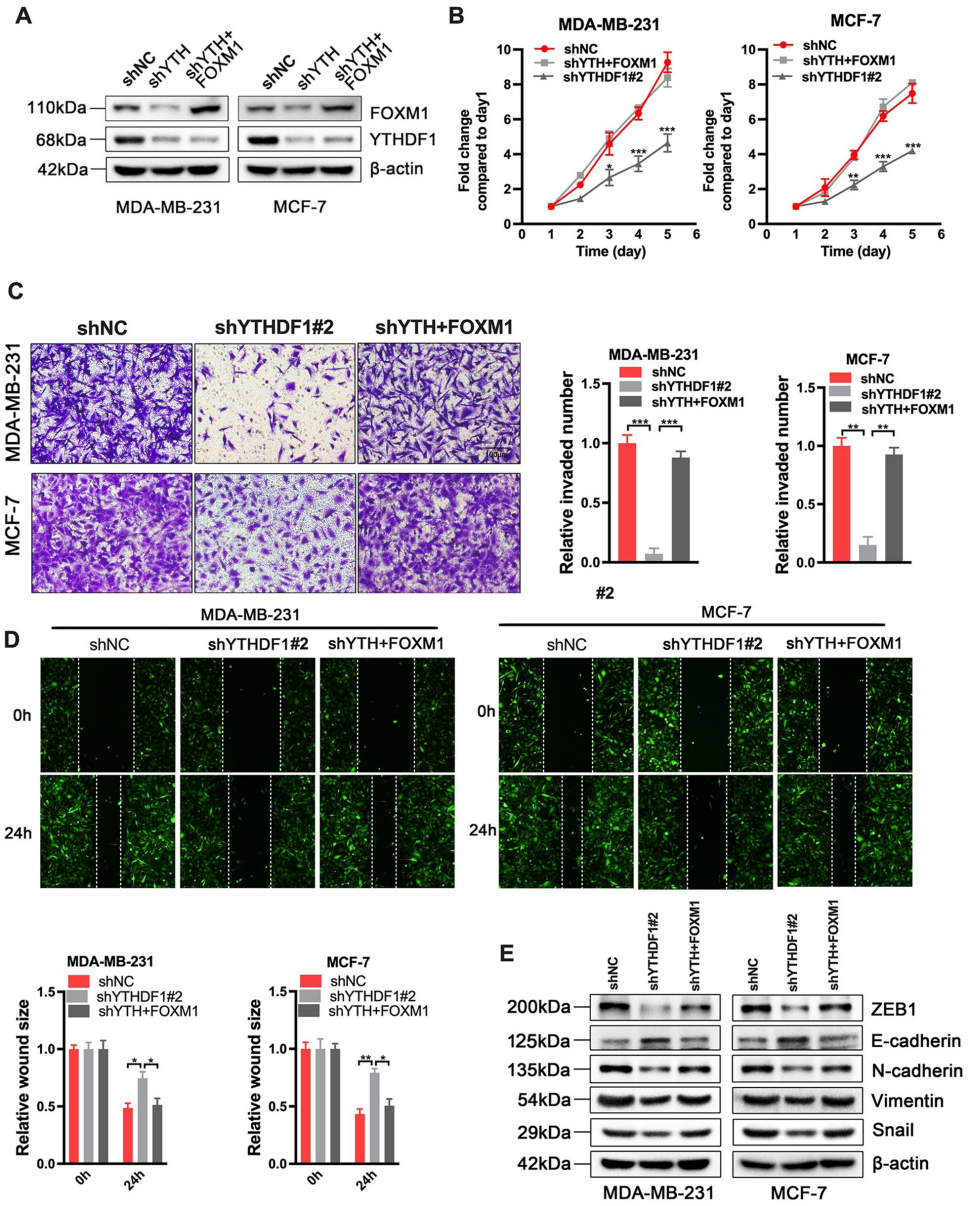
The anti-tumor effect of YTHDF1 silence could be abrogated by FOXM1 overexpression. **A** Western blot analysis for FOXM1 and YTHDF1 in MDB-MA-231 and MCF-7 cells with sh-YTHDF1-2 (shYTH) or sh-YTHDF1#2 + pcDNA-FOXM1 plasmid (shYTH + FOXM1) **B** CCK-8 assay, **C** Transwell invasion assay, **D** wound healing assay and **E** Western blot assay for EMT-related proteins in MDB-MA-231 and MCF-7 cells with shNC, sh-YTHDF1#2 (shYTH) or sh-YTHDF1#2 + FOXM1 (shYTH + FOXM1). All data were presented as means ± SD of at least three independent repetitions. Values are significant at *P < 0.05, **P < 0.01, ***P < 0.001 as indicated

We further verified the role of the YTHDF1/FOXM1 axis in breast cancer progression. Plasmids that contain the FOXM1 sequence (FOXM1) were transfected into the sh-YTHDF#2 MDA-MB-231 and MCF-7 cells, respectively. Western blot analysis was used to verify the transfection efficiency (Fig. 7A). Subsequently, CCK-8 assay, transwell, and wound healing assay were performed on breast cancer cells with sh-NC, sh-YTHDF1#2 or sh-YTHDF1#2 + FOXM1, respectively. The results showed that depletion of YTHDF1 inhibited tumor proliferation, migration, and invasion, which was partially abolished by overexpression of FOXM1 (Fig. 7B–D). Consistent with these results, the western blot assay validated that sh-YTHDF1#2 + FOXM1 cells could counteract the changes of epithelial and mesenchymal markers induced by YTHDF1 silence (Fig. 7E).

**Fig. 8:**
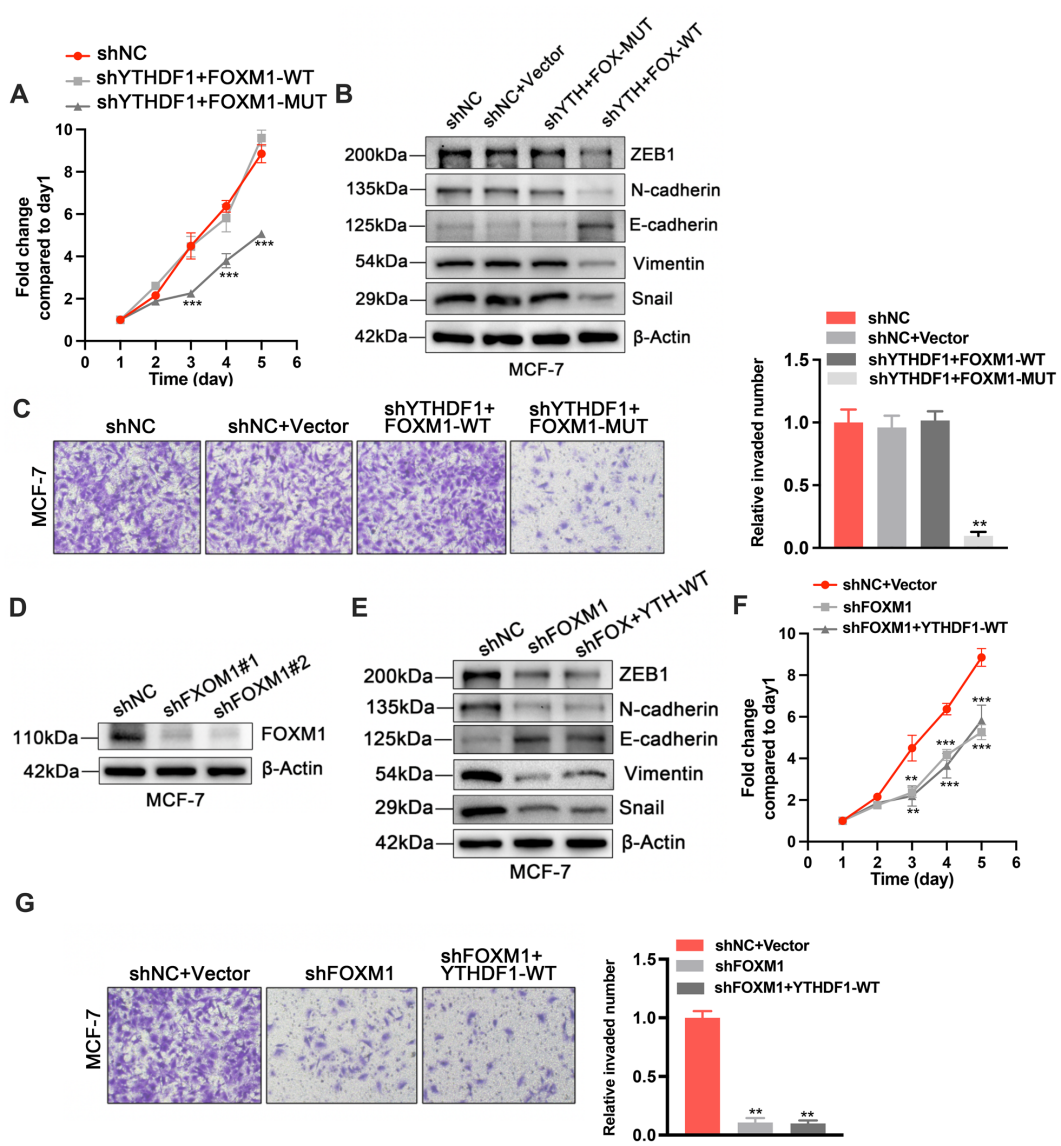
YTHDF1 overexpression could not reverse the anti-tumor effects caused by FOXM1 knockdown in MCF-7 cells. **A** CCK-8 assays in shNC, shYTHDF1#2 + FOXM1-WT and sh-YTHDF1#2 + FOXM1-MUT MCF-7 cells. **B** Western blots for EMT-related proteins and **C** Transwell invasion assays in shNC, shNC + Vector, sh-YTHDF1#2 + FOXM1-MUT and sh-YTHDF1#2 + FOXM1-WT MCF-7 cells. **D** FOXM1 protein expression in shNC, sh-FOXM1#1 and sh-FOXM1#2 transduced cells. **E** Western blots for EMT-related proteins, **F** CCK-8 assays and **G** Transwell invasion assays in shNC + Vector, sh-YTHDF1#2 + FOXM1-MUT and sh-YTHDF1#2 + FOXM1-WT MCF-7 cells. All data were presented as means ± SD of at least three independent repetitions. Values are significant at *P < 0.05, **P < 0.01, ***P < 0.001 as indicated

To further confirm that YTHDF1 regulates cell growth through FOXM1, we transfected FOXM1-MUT plasmid into sh-YTHDF1#2 MCF-7 cells and found that the mutant FOXM1 could not reverse the cell growth inhibition induced by YTHDF1 knockdown (Fig. 8A–C). The result confirmed that FOXM1 was downstream of YTHDF1. In addition, shRNAs were designed and transduced into MCF-7 cells to knock down FOXM1 expression (Fig. 8D). Through CCK-8, Transwell invasion and Western blot assays, we found that FOXM1 knockdown significantly inhibited cell growth, invasion and EMT. Besides, in FOXM1 knockdown MCF-7 cells, overexpression of YTHDF1 could not reverse the inhibitory effects on cell growth caused by FOXM1 knockdown (Fig. 8E–G).

Taken together, these data suggested that YTHDF1 knockdown blocks the proliferation, migration, and invasion of breast cancer cells, but that could be rescued by FOXM1 overexpression simultaneously.

## Discussion

m^6^A modification abnormalities have been shown to play critical roles in developing various tumors and are associated with the persistence of tumor stem cells [1]. In recent years, a study confirmed that YTHDF3 promotes breast cancer metastasis by promoting the translation of ST6GALNAC5, GJA1 and EGFR [27]. Another study demonstrated the demethylase FTO promotes breast cancer progression by inhibiting BNIP3 [28]. Ramesh et al. reported that high expression of YTHDF1 and TYHDF3 was closely associated with breast cancer metastasis and poor prognosis [21]. Despite the prognostic role of m6A “reader” YTHDF1 mentioned in the above article, however, the biomechanism of YTHDF1 involved in the carcinogenesis and metastasis of breast cancer remains unclear. Thus, it is urgent to elucidate the underlying mechanisms in breast cancer progression and discover reliable biomarkers to predict prognosis.

As an m^6^A reading protein, YTHDF1 promotes translation by recognizing and binding to the m^6^A modified mRNAs [29]. Several studies have explored its function. YTHDF1 could act as an oncogene or a tumor suppressor gene in tumors. YTHDF1 promoted the eIF3C translation in ovarian cancer, thus promoting the development and metastasis of ovarian cancer [25]. In lung cancer, YTHDF1 increased the translation of the YAP gene and induced metastasis and drug resistance in lung cancer cells [30]. In hepatocellular carcinoma (HCC), higher expression of YTHDF1 was associated with advanced stages and unfavorable prognosis [19]. Bioinformatic analysis indicated that in HCC, the expression of YTHDF1 was related to the expression of cell cycle-related genes. In addition, YTHDF1 promoted HCC EMT by upregulating Snail [31]. In colorectal cancer (CRC) cells, YTHDF1 induced cisplatin resistance by regulating glutamine metabolism through glutaminase [17]. In contrast to the tumor-promoting effects of YTHDF1 in the above tumors, YTHDF1 inhibited melanoma progression by promoting the translation of the tumor suppressor gene HINT2 [32].

In the present study, we found that the expression of YTHDF1 in breast cancer samples and breast cancer cell lines was significantly higher than that of the normal controls, and its high expression was significantly correlated with the shortened OS, larger tumor size, as well as positive lymphatic metastasis and distant metastasis. We explored the expression of YTHDF1 in different subtypes of breast cancer and did not find that it was significantly increased or decreased in the HER2+ subtype. However, its high expression predicted poor prognosis of HER2+ patients, suggesting that it might play an important role in helping to continuously activate HER2+ downstream signaling pathways (such as PI3K/AKT pathway) or alternative pathways (IGF-IR pathway) to induce resistance to HER2+ targeted therapy. After YTHDF1 was knocked down by shRNA in MCF-7 and MDA-MB-231 cells, the proliferation, migration, invasion and EMT of the tumor cells were significantly suppressed, and G0/G1 phase cell cycle arrest occurred. Subsequently, we confirmed that FOXM1 was a direct target of YTHDF1 through RIP, m^6^A-IP, YTHDF1-CLIP and polysome profiling analysis. YTHDF1 regulated FOXM1 mainly by facilitating its translation, which was implemented in a manner dependent on m^6^A.

EMT was considered an essential step in tumor metastasis [33], and its occurrence represents the transformation of differentiated epidermal cells into mesenchymal cancer cells [34]. It also plays a vital role in the occurrence, development and metastasis of breast cancer [35]. FOXM1 was a crucial gene that promoted EMT in a variety of tumors [36–40]. In breast cancer, FOXM1 was shown to promote EMT by facilitating Slug gene transcription [41]. FOXM1 expression and activity were known to be regulated in diverse ways. In breast cancer, HMGA1 [42], EGF [43, 44] and TGFβ [45, 46] signaling pathways, HIF-1α [47] all promoted EMT by upregulating FOXM1. In addition, FOXM1 could be epigenetic regulated [48] or deubiquitinated by USP21 [49] or OTUB1 [50]. In this study, we described a novel regulatory mechanism of FOXM1. That is, FOXM1 was modulated by YTHDF1 in an m^6^A-dependent manner. In addition, a previous study reported that FOXM1 not only promoted breast cancer progression and metastasis through EMT but also mediated PI3Kα inhibitor resistance in ER+ breast cancer [51].

Currently, most studies on YTHDF1 focused on regulating its target genes. Only a few studies have explored the regulation of YTHDF1 expression. For example, Hypoxia was found to upregulate YTHDF1 expression in a HIF-1α dependent manner in hepatocellular carcinoma [52]. c-Myc promoted the expression of YTHDF1 in colorectal carcinoma [53]. In addition, the Wnt/β-catenin signaling pathway [54], the m6a “eraser” ALKBH5 [55], and miR-3436 [56] were reported to regulate YTHDF1 expression in cancers. The regulatory mechanisms of YTHDF1 are complex. We will carry out further studies to explore the upstream of YTHDF1.

## Conclusion

We investigated the specific biological role of YTHDF1 in breast cancer, and our results revealed that YTHDF1 was aberrantly overexpressed and significantly associated with unfavorable survival rates of breast cancer. Moreover, we demonstrated that YTHDF1 played a crucial role in promoting cell proliferation and invasion in cancer cells via the YTHDF1/FOXM1 axis, aggravating breast cancer progression and metastasis consequently. Collectively, our study proposed a novel regulatory pathway of YTHDF1, which provided a new idea for breast cancer treatment and suggested that YTHDF1 may be a potential biomarker and therapeutic target for breast cancer patients.

## Supplementary Information

**Additional file 1: Figure S1.** (A) The expression of YTHDF1 in breast cancer patients of different stages. (B) Expression of YTHDF1 in breast cancer tissues based on breast cancer subclasses in the TCGA-BRCA dataset. The effects of YTHDF1 on overall survival (OS) in (C) Luminal, (D) HER2+ and (E) Basel-like breast cancer.

**Additional file 2: Figure S2.** Plasmid information of YTHDF1 and FOXM1. (A) Schematic image of YTHDF1-WT and YTHDF1-MUT plasmids (Flag-tagged). (B) The detailed mutation information of m^6^A motif in FOXM1-MUT plasmids.

**Additional file 3: Figure S3.** YTHDF1 knockdown by siRNAs inhibits the growth and invasive ability of MCF-7 cells. (A) Relative YTHDF1 mRNA expression levels after transfecting scramble si-NC, si-YTHDF1-1, si-YTHDF1-2 in MCF-7 cells. (B) CCK-8 assays, (C) Transwell invasion assays, (D) EdU proliferation assays and (E) Western blots for EMT-realted proteins in MCF-7 cells transfected with si-NC, si-YTHDF1-1 or si-YTHDF1-2. All data were presented as means ± SD of at least three independent repetitions. Values are significant at *P < 0.05, **P < 0.01, ***P < 0.001 as indicated.

**Additional file 4: Figure S4.** Polysome profiling assay in MCF-7 cells transduced with vector and YTHDF1-WT (Left) or YTHDF1-MUT (Right).

**Additional file 5: Table S1.** Primers for RT-qPCR. **Table S2.** Clinicopathological correlations of YTHDF1 expression in breast cancer.

## Data Availability

All data created or analyzed during this study are enrolled in this published article or are available from the corresponding author on reasonable request.

## Abbreviations

m^6^A: N6-methyladenosine
EMT: Epithelial-mesenchymal transformation
mRNA: Messenger RNA
CDS: Coding sequence
HE: Hematoxylin and eosin
CLIP: Cross-linking immunoprecipitation
m6A-IP: M6A immunoprecipitation
BRCA: Breast invasive carcinoma
OS: Overall survival
YTHDF1: YTH N6-methyladenosine RNA binding protein 1
