# Supplementary figures and images for: YTHDF1 promotes breast cancer progression by facilitating FOXM1 translation in an m6A-dependent manner
Source: Cell Biosci. 2022 Feb 23;12:19. doi: 10.1186/s13578-022-00759-w (PMC8867832; doi:10.1186/s13578-022-00759-w)

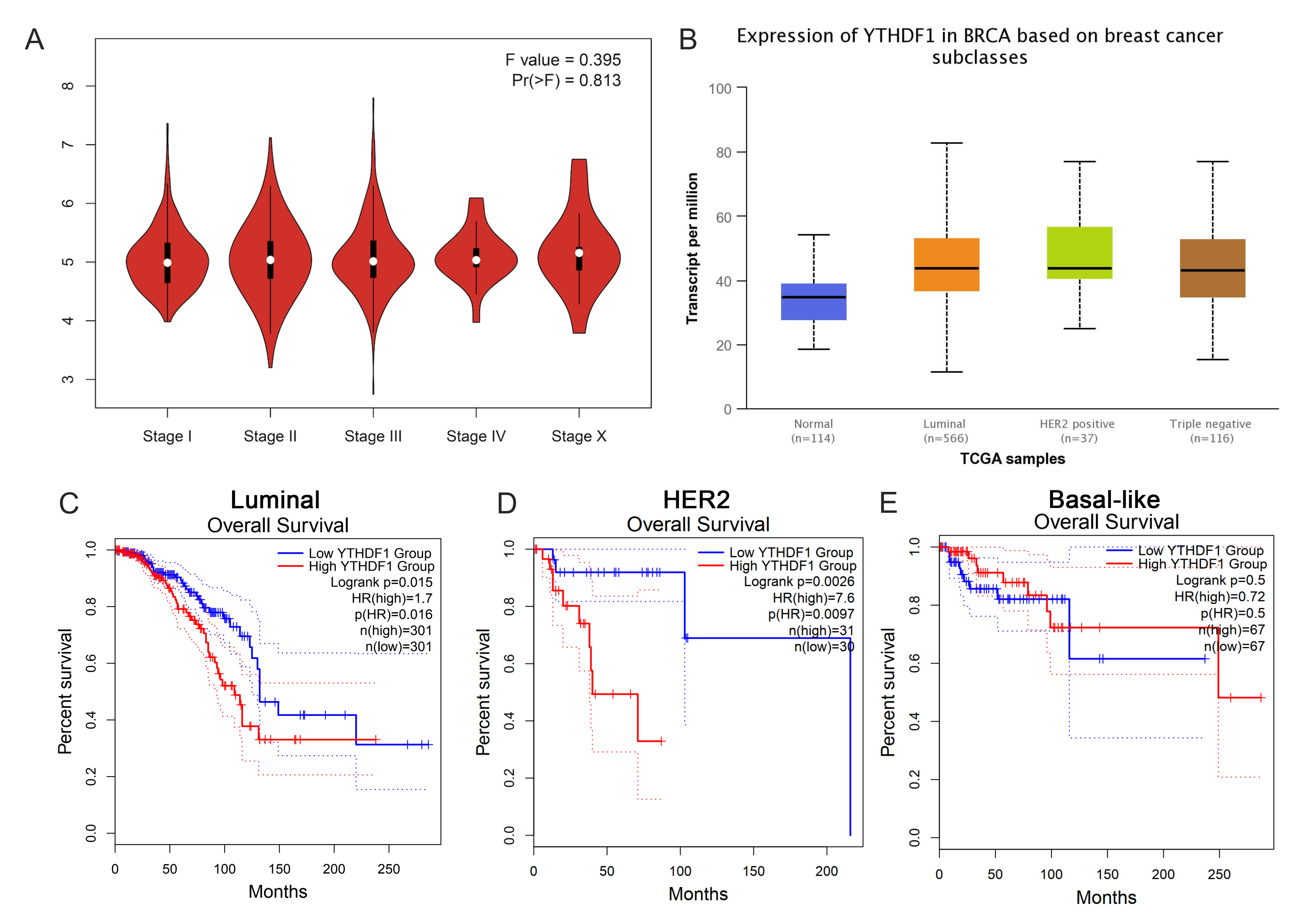

Supplement: Supplementary file 1 — Additional file 1: Figure S1. (A) The expression of YTHDF1 in breast cancer patients of different stages. (B) Expression of YTHDF1 in breast cancer tissues based on breast cancer subclasses in the TCGA-BRCA dataset. The effects of YTHDF1 on overall survival (OS) in (C) Luminal, (D) HER2+ and (E) Basel-like breast cancer. [file 13578_2022_759_MOESM1_ESM.tif]

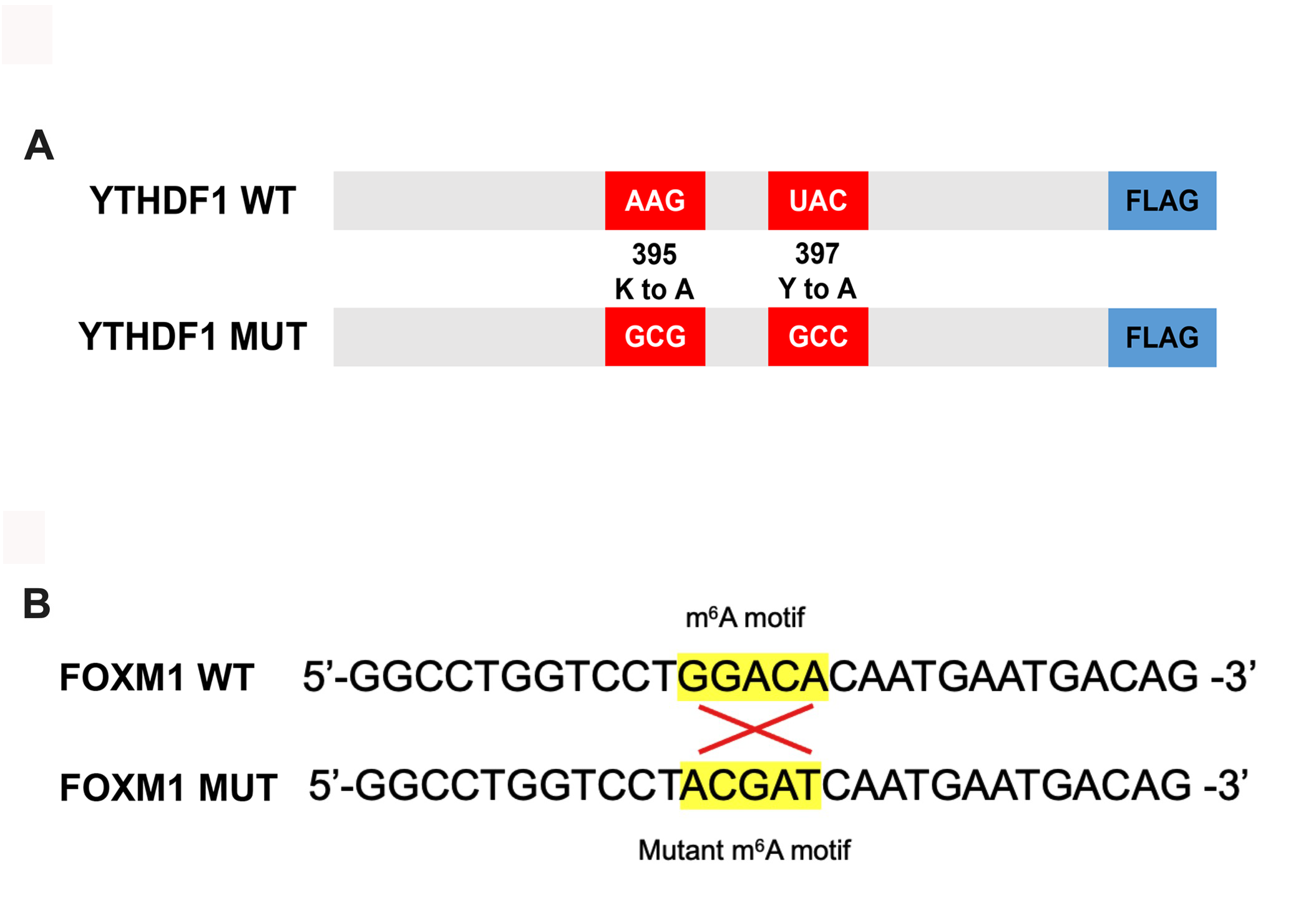

Supplement: Supplementary file 2 — Additional file 2: Figure S2. Plasmid information of YTHDF1 and FOXM1. (A) Schematic image of YTHDF1-WT and YTHDF1-MUT plasmids (Flag-tagged). (B) The detailed mutation information of m6A motif in FOXM1-MUT plasmids. [file 13578_2022_759_MOESM2_ESM.tif]

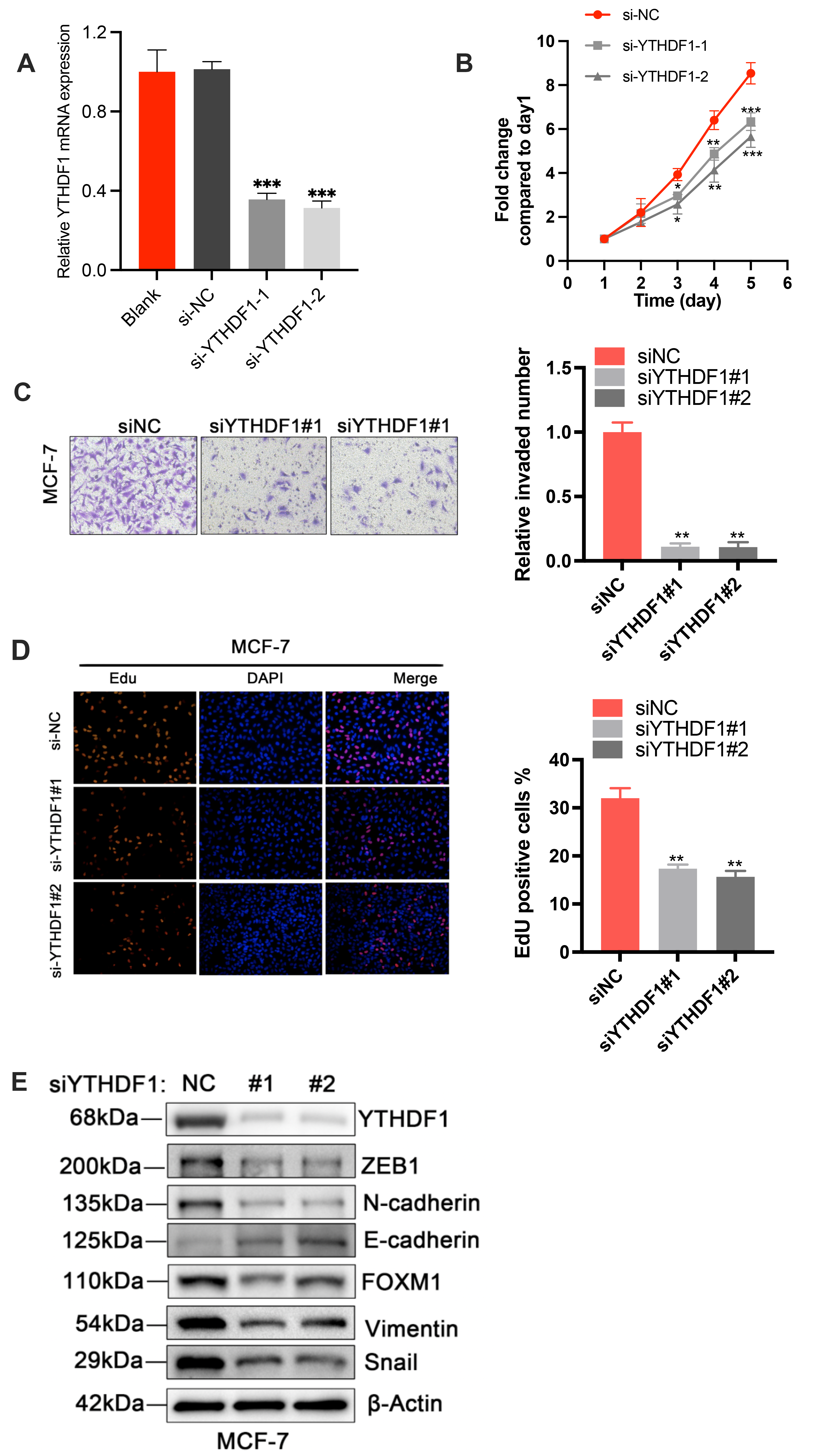

Supplement: Supplementary file 3 — Additional file 3: Figure S3. YTHDF1 knockdown by siRNAs inhibits the growth and invasive ability of MCF-7 cells. (A) Relative YTHDF1 mRNA expression levels after transfecting scramble si-NC, si-YTHDF1-1, si-YTHDF1-2 in MCF-7 cells. (B) CCK-8 assays, (C) Transwell invasion assays, (D) EdU proliferation assays and (E) Western blots for EMT-realted proteins in MCF-7 cells transfected with si-NC, si-YTHDF1-1 or si-YTHDF1-2. All data were presented as means ± SD of at least three independent repetitions. Values are significant at *P < 0.05, **P < 0.01, ***P < 0.001 as indicated. [file 13578_2022_759_MOESM3_ESM.tif]

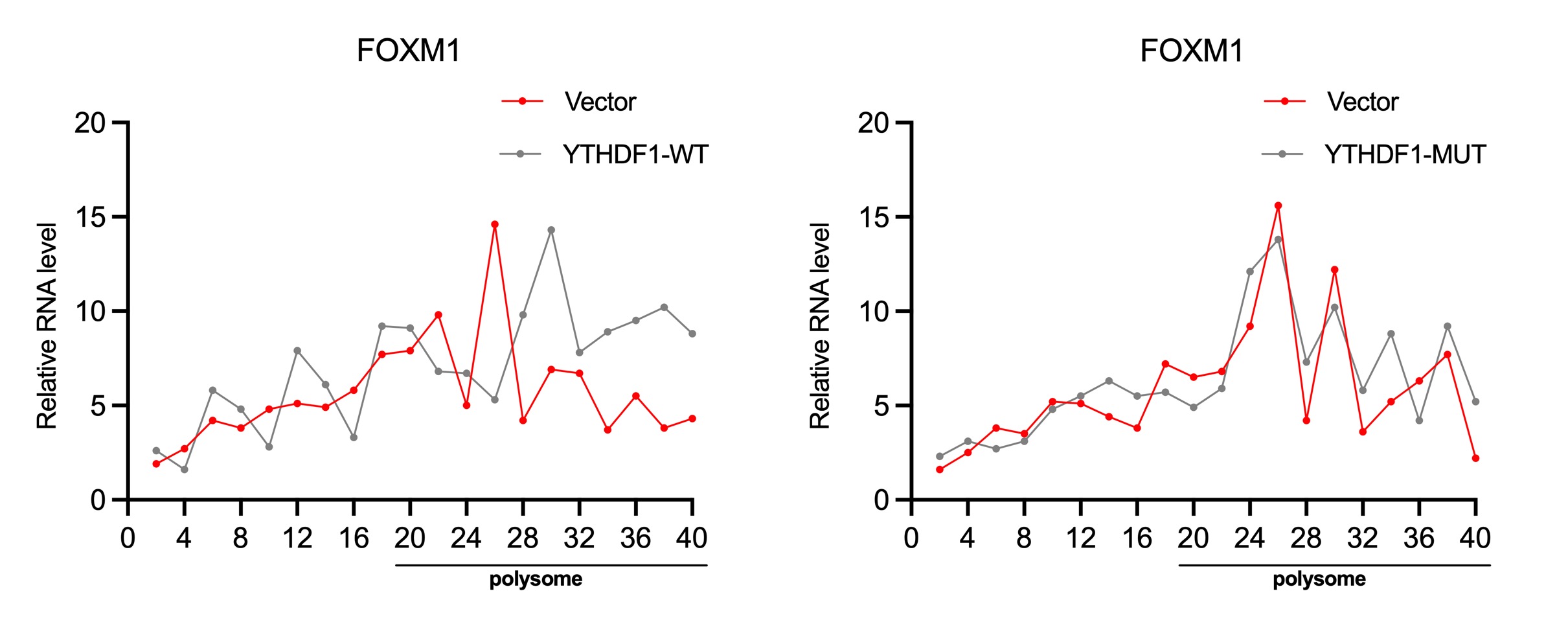

Supplement: Supplementary file 4 — Additional file 4: Figure S4. Polysome profiling assay in MCF-7 cells transduced with vector and YTHDF1-WT (Left) or YTHDF1-MUT (Right). [file 13578_2022_759_MOESM4_ESM.jpg]
