# Supplementary material for: YTHDF1 promotes breast cancer progression by facilitating FOXM1 translation in an m6A-dependent manner
Source: Cell Biosci. 2022 Feb 23;12:19. doi: 10.1186/s13578-022-00759-w (PMC8867832; doi:10.1186/s13578-022-00759-w)
Supplement: Supplementary file 5 — Additional file 5: Table S1. Primers for RT-qPCR. Table S2. Clinicopathological correlations of YTHDF1 expression in breast cancer. [file 13578_2022_759_MOESM5_ESM.docx]

Supplementary Table **S1. Primers for RT-qPCR.**

| Primers | Sequences |
| --- | --- |
| YTHDF1 | Forward: 5′- ACCTGTCCAGCTATTACCCG -3′ |
|  | Reverse: 5′- TGGTGAGGTATGGAATCGGAG -3′ |
| FZD5 | Forward: 5′- CATGCCCAACCAGTTCAACC-3′ |
|  | Forward: 5′- CGGCGAGCATTGGATCTCC -3′ |
| FZD7 | Forward: 5′- GTGCCAACGGCCTGATGTA -3′ |
|  | Forward: 5′- AGGTGAGAACGGTAAAGAGCG -3′ |
| FZD9 | Forward: 5′- TGCGAGAACCCCGAGAAGT -3′ |
|  | Forward: 5′- GGGACCAGAACACCTCGAC -3′ |
| WNT | Forward: 5’- ATTCTTGGTGGTCGCTAGGTA -3’ |
|  | Reverse: 5’- CGCCTTCTCCGATGTACTGC -3’ |
| CCNB1 | Forward: 5’- AATAAGGCGAAGATCAACATGGC -3’ |
|  | Reverse: 5’- AATAAGGCGAAGATCAACATGGC -3’ |
| ENY2 | Forward: 5’- TGAGAGCAGCGATTAACCAAAA -3’ |
|  | Reverse: 5’- AGTGTGCCTTCAACTGATCCT -3’ |
| SRC | Forward: 5’- ATTTCGAGACCCCTGACTCC -3’ |
|  | Reverse: 5’- ATCCTTGGGCTTGCTCTTG -3’ |
| FOXM1 | Forward: 5’- CTTCGGAGCTACGGCCTAA -3’ |
|  | Reverse: 5’- GACGGGGGCTAGTTTTCATT -3’ |
| CCNE1 | Forward: 5’- AGAAATGGCCAAAATCGACA -3’ |
|  | Reverse: 5’- CCCGGTCATCATCTTCTTTG -3’ |
| CCNE2 | Forward: 5’- GGAACAAGTGATTTCTCCAGATT -3’ |
|  | Reverse: 5’- TGTCATGAACATATCTGCTCTCC -3’ |
| ASNS | Forward: 5’- GTAGTTGACCCGCTGTTTGG -3’ |
|  | Reverse: 5’- TCACCATCCACTTTGGTCTG -3’ |
| EIF4BP1 | Forward: 5’- TCTGAGGAAGGACCAGCAAG -3’ |
|  | Reverse: 5’- GATCCTTTTTGCCATTTTCC -3’ |

Supplementary Table S2. Clinicopathological correlations of YTHDF1 expression in breast cancer.

|  | **Expression of YTHDF1** | | | ***p*-value** |
| --- | --- | --- | --- | --- |
|  | **Low** | **High** | **total** |  |
| Age(y) |  |  |  |  |
| ≤ 60 | 24 | 33 | 57 | 0.934 |
| > 60 | 27 | 36 | 63 |  |
| Tumor size |  |  |  |  |
| ≤ 2 | 17 | 8 | 25 | 0.005** |
| > 2 and ≤ 5 | 26 | 42 | 68 |  |
| > 5 | 8 | 19 | 27 |  |
| Tumor differentiation |  |  |  |  |
| Well | 12 | 14 | 26 | 0.940 |
| Moderate | 15 | 24 | 39 |  |
| Poor | 24 | 31 | 55 |  |
| TNM stage |  |  |  |  |
| I~II | 22 | 31 | 53 | 0.847 |
| III~IV | 29 | 38 | 67 |  |
| Lymphatic metastasis |  |  |  |  |
| Positive | 31 | 57 | 88 | 0.007** |
| Negative | 20 | 12 | 32 |  |
| Distant metastasis |  |  |  |  |
| Positive | 28 | 51 | 69 | 0.03 * |
| Negative | 23 | 18 | 41 |  |
